# Role of an external electric field on hybrid halide perovskite CH_3_NH_3_PbI_3_ band gaps

**DOI:** 10.1038/s41598-018-29935-0

**Published:** 2018-08-21

**Authors:** Denghui Ji, Mula Na, Shuling Wang, Hong Zhang, Kun Zhu, CongMin Zhang, Xiuling Li

**Affiliations:** 1 School of Electrical engineering, Liupanshui Normal University, Liupanshui City, 553004 People’s Republic of China; 2 School of Mathematics and Physics, Hebei University of Engineering, Handan City, 056038 People’s Republic of China; 3 Department of General Education Courses, Hebei Agricultural University Bohai Campus, Huanghua City, 061100 People’s Republic of China; 4 College of Physics and Information Engineering, Hebei Advanced Thin Films Laboratory, Hebei Normal University, Shijiazhuang City, 050024 People’s Republic of China

## Abstract

The organic-inorganic perovskite CH_3_NH_3_PbI_3_ has attracted much attention due to their power conversion efficiency as a potential photovoltaic material, but the role of an external electric field has not been well understood. Based on first-principles calculations, the effects of an external electric field (*E*) applied along the [111] direction of the orthorhombic perovskite, CH_3_NH_3_PbI_3_, on its electronic structure and optical properties are investigated. Our results indicate that the electric field strength affects the band gap (*E*_g_) of CH_3_NH_3_PbI_3_ (MAPbI_3_, MA = CH_3_NH_3_). The energy difference between the two peaks closest to the Fermi level in the density of states diagram decreases with increasing applied electric field strength along the [111] direction, indicating that the covalent character increases between A-sites cations and I-sites anions. Both the cell volume and the final energy show the same increasing trend. The absorption peaks move toward the visible-frequency range, with the optimal band gap of 1.1–1.45 eV and *E* = 0.04–0.06 eV/Å/e. In particular, the non-linear change of the second-order Stark effect causes a non-linear change in the band gap.

## Introduction

The organic-inorganic hybrid perovskite CH_3_NH_3_PbI_3_ has attracted much attention for its use as a photon absorber in thin-film solar cells. In addition, its synthesis is relatively straightforward and can be achieved by simple layer deposition^1–6^. Although great success has been achieved in improving the photovoltaic energy conversion efficiency of this perovskite^2,7^, perovskites are ideal because they absorb most of the solar spectrum, from the ultraviolet region to the near-infrared region, generate more excitons as more photons absorbed, and bring the energy levels closer to that of the electron transport layer (ETL) or hole transport layer (HTL). The optimal band gap for a single junction solar cell is between 1.1 and 1.4 eV^8,9^. However, CH_3_NH_3_PbI_3_ has a narrow direct band gap energy (*E*_g_) of 1.51 eV, which is not optimal and hinders its application.

Using first-principles calculations, Amat *et al*.^10^ investigated tetragonal APbI_3_ perovskites with A = Cs^+^, (CH_3_NH_3_)^+^, and (NH_2_)_2_CH^+^ and found that CH_3_NH_3_PbI_3_ and (NH_2_)_2_CHPbI_3_ have the same band gap. Hao *et al*.^11^ studied the performance of CH_3_NH_3_Sn_1−*x*_Pb_*x*_I_3_ perovskite solar cells. Their results indicated that the band gaps of mixed Pb/Sn hybrid perovskites have two extremes, 1.55 and 1.35 eV, depending on the ratio of Pb to Sn, but the band gaps were narrow (<1.3 eV). Furthermore, a band gap of 1.60 eV for CH_3_NH_3_PbI_3_^12^, 2.39 eV for CH_3_NH_3_PbBr_3_, and 3.17 eV for CH_3_NH_3_PbCl_3_^13,14^ was obtained. Moreover, it was shown that the optical band gap can be tuned from a direct band gap of 1.52 eV to an indirect band gap of 2.64 eV by varying the CH_3_NH_3_I concentration^15^. In addition, the band gap of the tetragonal phase of CH_3_NH_3_PbI_3_ decreased with decreasing temperature^16^, following the relationship *E*_g_ (*T*) = *E*_g_ (*T*_0_) − *b*.*k*_B_.(*T*_0_ − *T*)^2^. Thus, finding the optimal band structure remains an important physical problem.

In many cases, the electric field effect can change the geometric, electronic, magnetic, and band structures of materials, and thus regulate their physical properties. Zhao *et al*.^17^ reported the influence of external electric fields on the electronic structure and optical properties of TiO_2_. They found that the band gap of TiO_2_ becomes narrower with increasing electric field strength, decreasing to 0 eV when the electric field is 0.25 eV. Varignon *et al*.^18^ used an electric field to control the Jahn-Teller distortions in bulk perovskites such as SrTiO_3_, BaMnO_3_, YMnO_3_, and BiFeO_3_. Bellaiche *et al*. reported that an external electric field could induce polarization paths in PbZr_1−*x*_Ti_*x*_O_3_ perovskites and lead to the expected sequence of tetragonal, A-type monoclinic, and rhombohedral structures^19^. Xu *et al*.^20^ demonstrated that electric fields could induce a change from a ferroelectric phase to an antiferroelectric phase in a lead-free NaNbO_3_-based polycrystalline ceramic. Therefore, it is very important and valuable to investigate the relationship between external electric fields and the physical properties of the organic-inorganic hybrid perovskite CH_3_NH_3_PbI_3_. CH_3_NH_3_PbI_3_ undergoes two phase transitions, one at 160 K (orthorhombic to tetragonal) and the other at 330 K (tetragonal to cubic)^21^. Using Density functional theory (DFT) calculations, Leppert *et al*. investigated the Rashba effect induced by the electric field and strained in the hybrid halide perovskite CH_3_NH_3_PbI_3_ with a tetragonal and cubic structure^22^. Although the orthorhombic phase is realized with rotations about the C–N axis that freeze out when *T* ≤ 162 K, temperature is not the only factor affecting these properties. An external electric field or magnetic field could also be an important factor, so we chose the orthorhombic CH_3_NH_3_PbI_3_ as our subject to investigate these effects.

In this article, we investigate the effects of an external electric field applied along the [111] direction on the geometry structure, electronic energy band structure, total density of states, and optical properties of CH_3_NH_3_PbI_3_. This study provides a method for obtaining the optimal band gap of CH_3_NH_3_PbI_3_ and expands the scope of its applications.

## Results and Discussion

The effects of the external electric field (*E*) direction, including the [001], [010], [100], [110], and [111] directions, on the band structure of CH_3_NH_3_PbI_3_ were studied. The application of an electric field along the [111] direction in CH_3_NH_3_PbI_3_ decreases the band gap, while fields aligned along the other directions increase the band gap. Therefore, we only investigated the physical properties of CH_3_NH_3_PbI_3_ under an electric field aligned along the [111] direction to obtain the optimal band gap of 1.1–1.4 eV.

### Band structure of CH_3_NH_3_PbI_3_ under the external electric field

**Figure 1:**
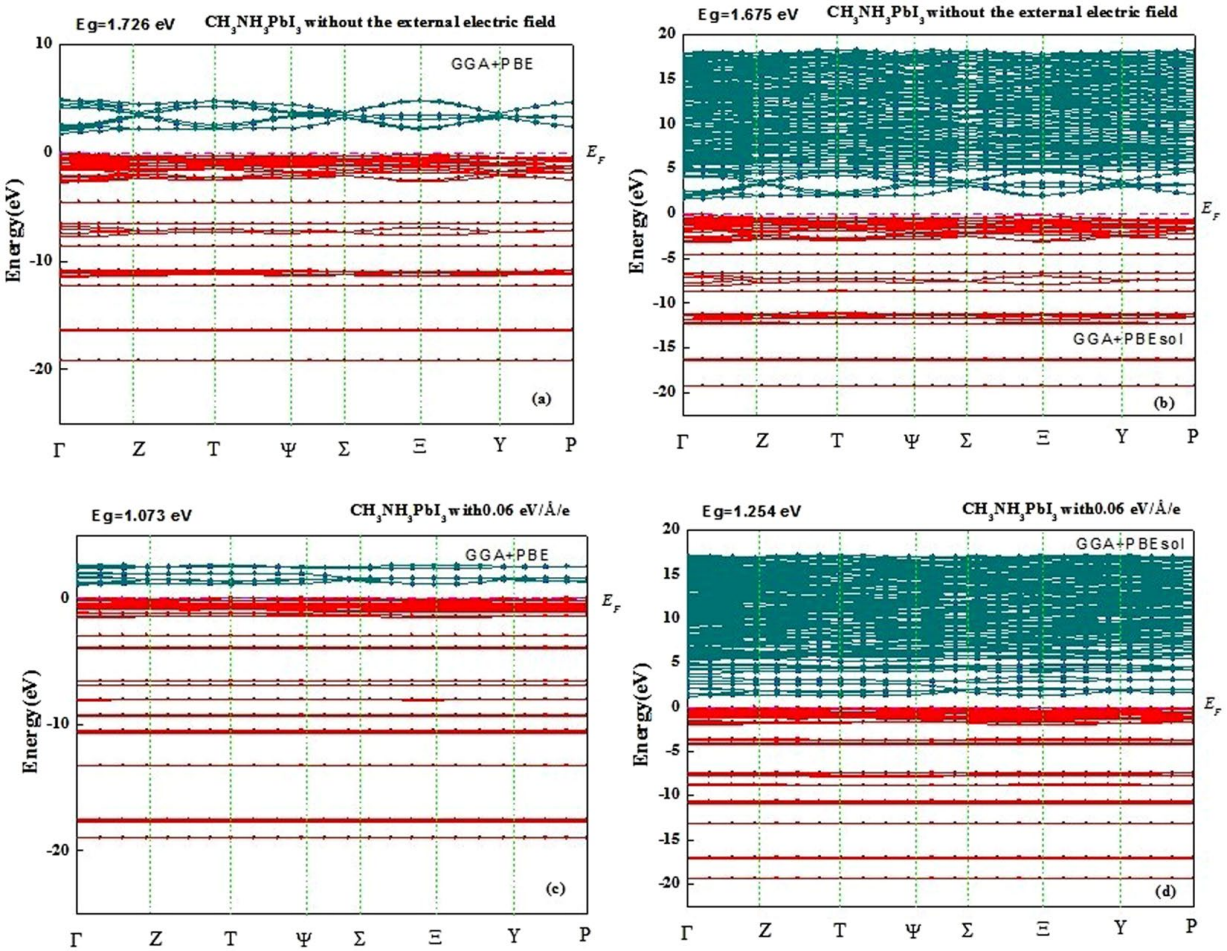
Band structure of CH_3_NH_3_PbI_3_, calculated by GGA + PBE without the external electric field (**a**), calculated by GGA + PBEsol without the external electric field (**b**), calculated by GGA + PBE with the external electric field 0.06 eV/Å/e (**c**), and calculated by GGA + PBEsol with the external electric field 0.06 eV/Å/e (**d**).

The band structure of CH_3_NH_3_PbI_3_ in the absence of an external electric field is shown in Fig. 1 (a,b). When the external electric field is equal to zero, the valence band maximum (VBM) and the conduction band minimum (CBM) are located at the same Γ-point, which indicates that CH_3_NH_3_PbI_3_ possesses direct semiconductor characteristics. The calculated band is 1.726 eV and 1.675 eV, corresponding to the generalized gradient approximation (GGA) functional developed by Perdew, Burke, and Ernzerhof PBE^23^ and PBEsol^24^, which are similar to the results^25^ reported by Menéndez-Proupin *et al*. The detailed calculation methods were shown in the Method section. It should be noted that the number of conduction bands calculated using GGA + PBEsol is greater than that calculated using GGA + PBE. In the presence of an applied external electric field with a strength ranging from 0.01 to 0.06 eV/Å/e, the band structures of the orthorhombic CH_3_NH_3_PbI_3_ are similar. Figure 1 (c,d) show the band structure when *E* = 0.06 eV/Å/e. The conduction band shifts downward to the Fermi level (0 eV) and the energy band near the CBM is more dispersed, resulting in a change in the band gaps. All the configurations indicate an indirect band gap semiconductor, and the VBM and CBM are located at the centre of the Γ-point in *k* space. Because of the narrower indirect band gap of the CH_3_NH_3_PbI_3_ semiconductor, only a small amount of energy is required for the formation of excitons. However, the GGA + PBE and GGA + PBEsol methods underestimate the band gap because of self-interaction errors, suggesting that the actual band gap of CH_3_NH_3_PbI_3_ is slightly smaller than the calculated values.

**Figure 2:**
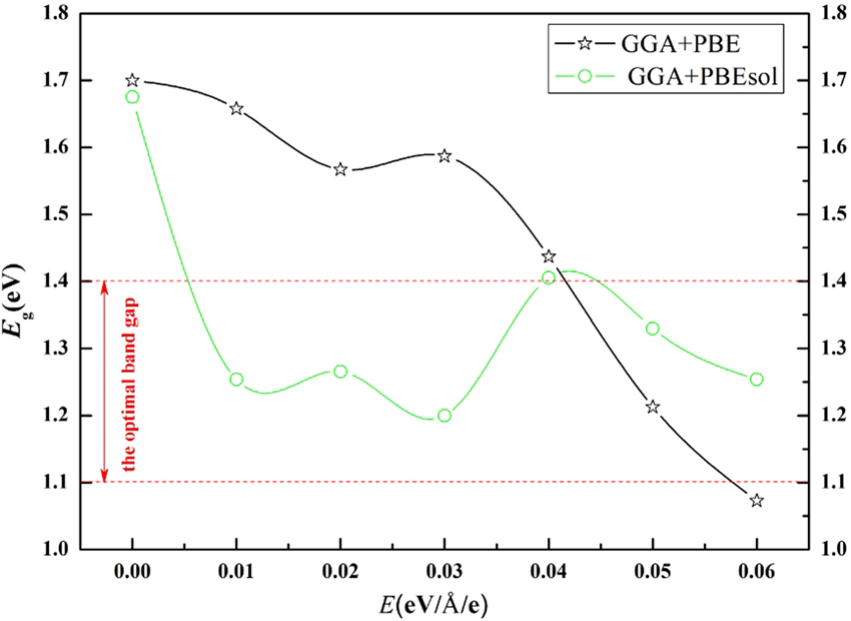
The curves of band gap (*E*_g_) versus the external electric field (*E*) of CH_3_NH_3_PbI_3_ perovskite calculated by GGA + PBE and GGA + PBEsol.

The band gaps of CH_3_NH_3_PbI_3_ are strongly affected by the applied electrical field strength. As the external electric field strength increases, the band gaps calculated by GGA + PBE first decrease linearly, then increase slightly, and finally, decrease linearly. The band gaps calculated by GGA + PBEsol indicated the optimal value between 1.1 and 1.4 eV for all the structures under an external electric field, as shown in Fig. 2. Therefore, both the results indicated that the external electric field contributes to a decrease in the band gap.

### Density of states of CH_3_NH_3_PbI_3_ under an external electric field

**Figure 3:**
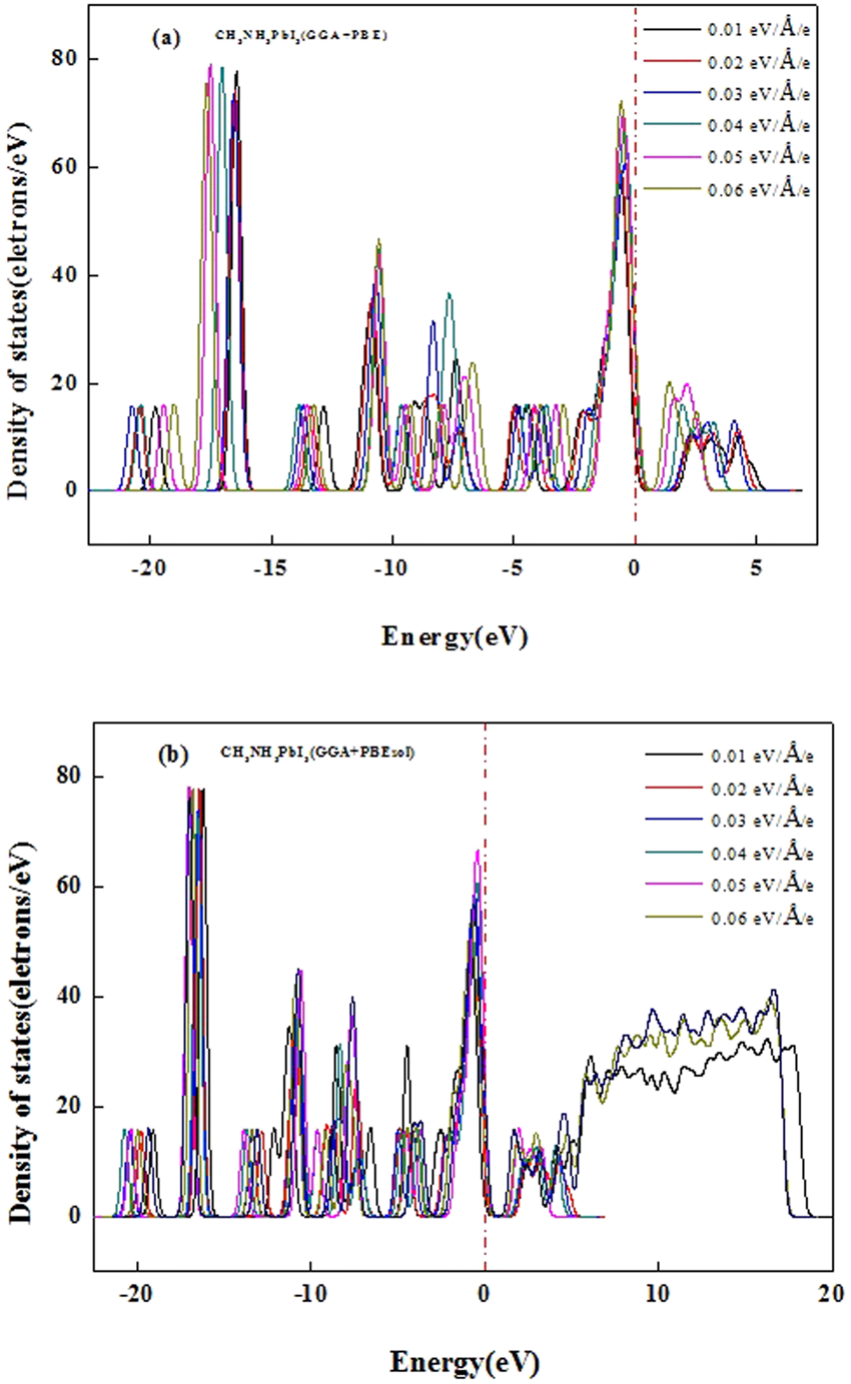
The total density of states (TDOS) of CH_3_NH_3_PbI_3_ calculated by GGA + PBE or GGA + PBEsol with the external electric field *E = *0.00–0.06 eV/Å/e.

Figure 3 (a) calculated by GGA + PBE and (b) calculated by GGA + PBEsol show the total density of states (TDOS) of CH_3_NH_3_PbI_3_ in the presence of an external electric field (*E* = 0.00–0.06 eV/Å/e). As the external electric field increases, (i) the two peaks corresponding to orbital energies from −22.5 to −15 eV gradually broaden and then become narrow, reaching their maximum widths when *E* = 0.03 eV/Å/e. (ii) The five peaks corresponding to orbital energies between −15 and −5.5 eV gradually broaden. (iii) Parts of the TDOS of the orbitals crossing the Fermi level increase with increasing external electric field, suggesting that the band gap of CH_3_NH_3_PbI_3_ decreases. (iv) The CBM gradually narrows and shifts to the Fermi level, which also decreases the band gap, suggesting a pseudo-energy gap decrease, while the strength of the covalent character increases between the MA-cations and I anions.

**Figure 4:**
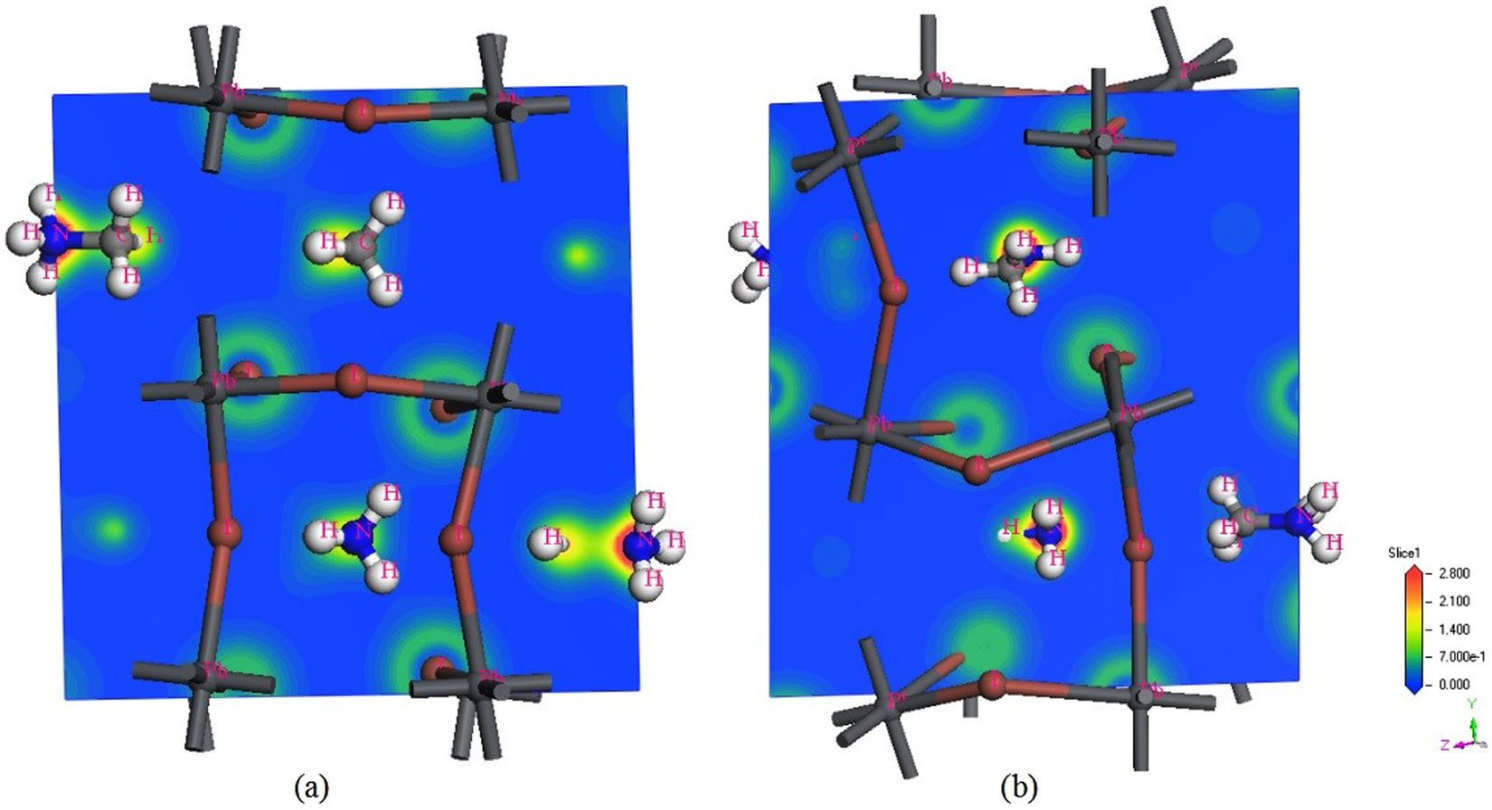
The electron density of CH_3_NH_3_PbI_3_ calculated by GGA + PBEsol with the external electric field 0.00 eV/Å/e (**a**), and 0.06 eV/Å/e (**b**).

The electron densities of CH_3_NH_3_PbI_3_ when *E* = 0.00 and 0.06 eV/Å/e were shown in Fig. 4 (a,b), calculated by GGA + PBEsol. It can be seen that: (i) the lost electronic ions are H and Pb cations, and the gain electronic ions are C, N and I anions. (ii) With increasing external electric field, the electron density of C-N increases, thus increasing the strength of the associated covalent bond. (iii) The exact value of gain or lost electrons can be obtained based on Milliken Charge Analysis Method of Wave Function. For the *E* = 0.00, the lost average H cations have three kinds including 0.34, 0.22, and 0.21 electrons, the lost average Pb cations have one kind with 0.88 electrons, the gain anions with C and N have 0.61 and 0.70 electrons, and the gain I anion have two kinds including 0.36 and 0.52 electrons. For the *E* = 0.06 eV/Å/e, the lost average H cations have seven kinds including 0.15, 0.16, 0.29, 0.30, 0.34, 0.35 and 0.44 electrons, the lost average Pb cations have one kind with 0.54 electrons, the gain C anions have 0.65 electrons, the gain N anions have two kinds including 0.77 and 0.76 electrons, and the gain I anion have six kinds including 0.29, 0.37, 0.38, 0.39, 0.40, and 0.41 electrons.

**Figure 5:**
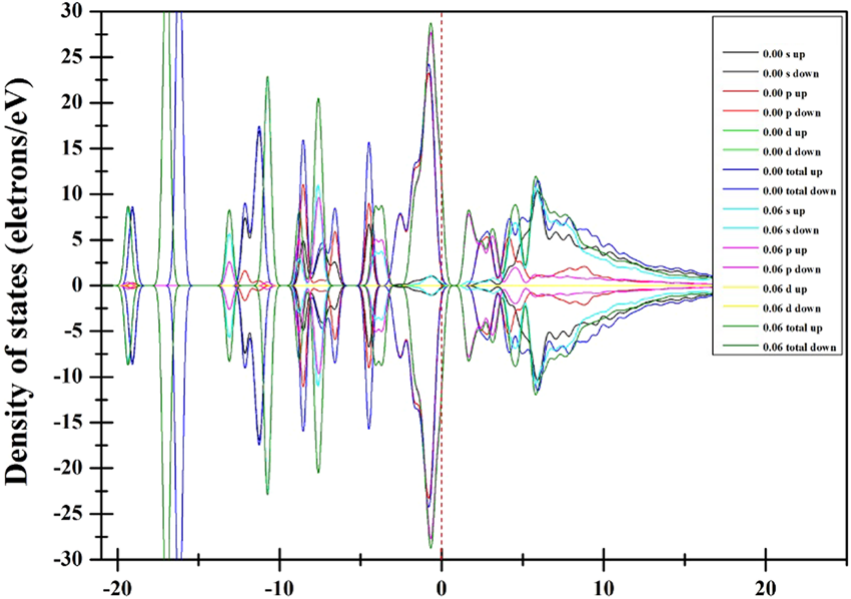
The partial-total density of states (PDOS) of CH_3_NH_3_PbI_3_ (GGA + PBEsol) with the external electric field *E = *0.00 and 0.06 eV/Å/e.

Figure 5 shows the partial density of states (PDOS) of CH_3_NH_3_PbI_3_ (GGA + PBEsol) under applied external electric field strengths of *E* = 0.00 and 0.06 eV/Å/e. The electronic orbitals 5d6s6p, 5s5p, 2s2p, 2s2p, and 1s are modeled as the valence orbitals for Pb, I, C, N, and H, respectively. The s-p hybrid level increases with external electric field increasing, and the effect on the conduction band is much more pronounced than that on the valance band. The peak near the orbital energy level at −5.0 eV splits into two peaks, which is attributed to the Stark effect of the s and p electrons, and one peak position shifts to the Fermi level. The external electric field causes the d electrons of Pb to shift to higher energies, but the d electrons do not affect the s and p electrons.

The Stark effect can induce the splitting of degenerate energy levels. The degree of the band splitting increases with the electric field, and a smaller band gap can be obtained. All peaks of the orbital energy level in the DOS curves broaden and shift to lower energies as *E* increases. The physical mechanisms underlying the change in the band structure of CH_3_NH_3_PbI_3_ is ascribed to the C-N and Pb-I bond lengths, changes in the lattice structure, and charge transfer between the Pb and I atoms. The lattice distortion caused by the mutation of lattice parameters may lead to change in the microscopic electric structure, band structure, and density of states, similar to the results reported in ref.^17^.

### Optimized structure of CH_3_NH_3_PbI_3_ under an external electric field

**Figure 6:**
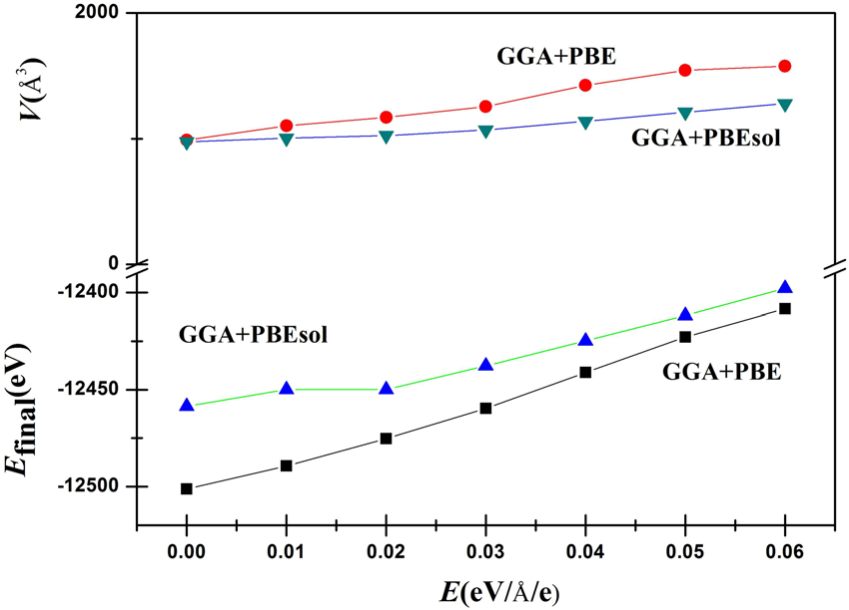
The curve of the final energy (*E*_final_) and cell volumes (*V*) increases with the external electric field *E*.

**Figure 7:**
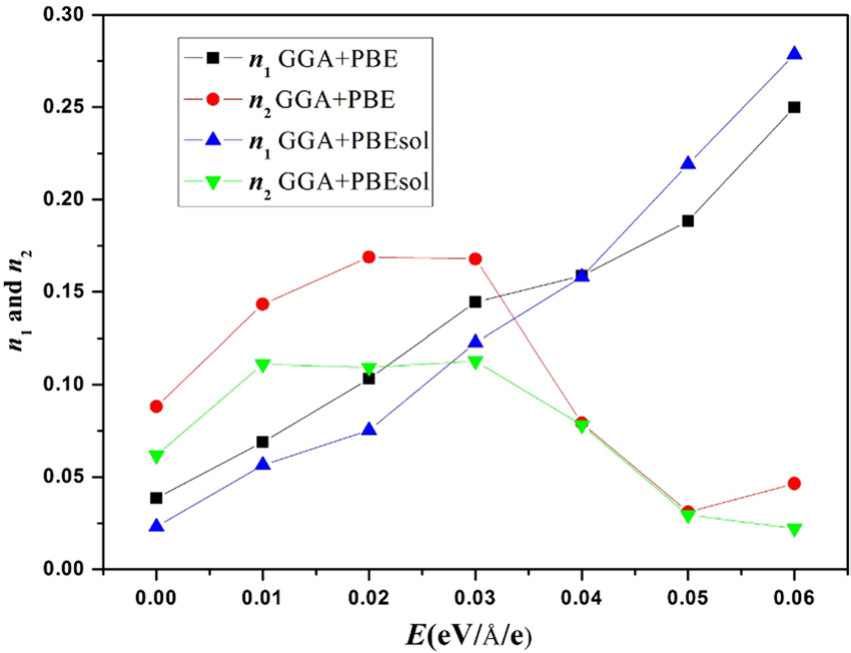
Parameters *n*_1_ and *n*_2_ *versus* the external electric field *E*. Here ${n}_{1}=\frac{b\,/\,\sqrt{2}-a}{a}$, and ${n}_{2}=\frac{b\,/\,\sqrt{2}-c}{c}$.

The crystal structure determines the physical properties of the material; the lattice parameters (*a*, *b*, and *c*), cell volume (*V*), bond length (*d*), and final energies (*E*_Final_) are listed in Table 1. The external electric field applied along the [111] direction stretches the lattice parameters. Figure 6 shows that both the final energies and the cell volumes increase monotonically, and the final energies obtained by GGA + PBE are lower than those obtained by GGA + PBEsol. The GGA + PBE results follow the fitted relationships *E*_Final_ (*E*) = 1591.989*E* – 12504.565 and *V* (*E*) = 1035.804*E* + 983.666. In addition, the bond distances, including the *d*(C-N) and *d*(I-Pb) distances, increase monotonously with increasing *E*, which leads to weak interactions between C and N as well as between I and Pb. To illustrate the extent of lattice distortion, two parameters (${n}_{1}=\frac{b\,/\,\sqrt{2}-a}{a}$, and ${n}_{2}=\frac{b\,/\,\sqrt{2}-c}{c}$) can be used for describing the degree of deviation from the cubic structure^26^. The curves for these parameters *versus* the external electric field are shown in Fig. 7. For the results based on the GGA + PBE or GGA + PBEsol method, both *n*_1_ and *n*_2_ are positive, suggesting that the orthogonal structures are of the O type, where $\frac{c}{\sqrt{2}} > b$, $\frac{a}{\sqrt{2}} > b$, and the deviations range from 2.31% to 27.86%, which are much greater than those for single inorganic perovskites^27^.

**Table 1:** Optimized structure of CH_3_NH_3_PbI_3_ with the external electric field *E* = 0.00–0.06 eV/Å/e including lattice parameters (*a*, *b*, *c*), bond distances (*d*), cell volumes (*V*), the final energies (*E*_Final_), Cartesian coordinates of Pb, C, and N, and the Pb-N-C angle. Units are Å, Å, Å^3^, eV, 1, and °.

Functions	Optimized structure	*x* = 0.00	*x* = 0.01	*x* = 0.02	*x* = 0.03	*x* = 0.04	*x* = 0.05	*x* = 0.06
PBE	*a*	8.9032	9.2071	9.2584	9.2498	9.3200	9.2692	9.0738
	*b*	13.0752	13.9164	14.4428	14.9694	15.2694	15.5758	16.0369
	*c*	8.4972	8.6082	8.7385	9.0650	10.0066	10.6834	10.8378
	*V*	989.1600	1102.9588	1168.4963	1255.1677	1424.0542	1542.4216	1577.0738
	*d*(C-N)	1.493	1.488	1.490	1.493	1.496	1.500	1.502
	*d*(I-Pb)	3.169	3.273	3.430	3.538	3.690	3.773	4.318
		3.320	3.539	3.634	3.761	3.874	4.012	4.144
	*E*_Final_	−12501.21889	−12489.29171	−12475.24448	−12459.64554	−12441.06694	−12422.84539	−12408.32328
	Cartesian coordinates (*x*,*y*,*z*)							
	Pb	(0,0,0.5)	(0,0,0.5)	(0,0,0.5)	(0,0,0.5)	(0,0,0.5)	(0,0,0.5)	(0,0,0.5)
	C	(0.0876,0,75, 0.9447)	(0.0866,0.75, 0.9556)	(0.0932,0.75, 0.9159)	(0.0994,0.75, 0.9211)	(0.0994,0.75, 0.9211)	(0.1094,0.75, 0.9107)	(0.1152,0.75, 0.9041)
	N	(−0.0585, 0.75, 1.0185)	(−0.0651, 0.75, 1.013)	(−0.0493, 0.75, 1.0065)	(−0.0429, 0.75, 0.9990)	(−0.0429, 0.75, 0.9890)	(−0.0285, 0.75, 0.9843)	(−0.0229, 0.75, 0.9797)
	*θ*	77.005	78.465	76.549	78.138	77.338	76.718	76.674
PBEsol	*a*	8.8774	8.9074	8.8567	8.7382	8.6475	8.4017	8.2677
	*b*	12.8425	13.3065	13.4663	13.8718	14.16068	14.4834	14.9474
	*c*	8.5542	8.4694	8.5846	8.8155	9.2884	9.9500	10.3411
	*V*	975.2591	1003.8505	1023.8576	1068.5584	1137.4005	1210.7628	1277.9559
	*d*(C-N)	1.481	1.479	1.481	1.482	1.482	1.479	1.472
	*d*(I-Pb)	3.201	3.388	3.413	3.492	3.578	3.700	3.856
		3.299	2.952	3.004	2.990	3.513	3.545	3.350
	*E*_Final_	−12458.46453	−12449.90457	−12449.90457	−12437.68499	−12424.7522	−12411.71698	−12397.74163
	Cartesian coordinates (*x*,*y*,*z*)							
	Pb	(0,0,0.5)	(0,0,0.5)	(0,0,0.5)	(0,0,0.5)	(0,0,0.5)	(0,0,0.5)	(0,0,0.5)
	C	(0.0905,0,75, 0.9352)	(0.0947,0.75, 0.9333)	(0.0968,0.75, 0.9226)	(0.1019,0.75, 0.9160)	(0.1049,0.75, 0.9130)	(0.1100,0.75, 0.9072)	(0.1142,0.75, 0.8987)
	N	(−0.0600, 0.75, 1.0093)	(−0.0549, 0.75, 1.0089)	(−0.0484, 0.75, 1.0080)	(−0.0406, 0.75, 1.0073)	(−0.0351, 0.75, 1.0050)	(−0.0246, 0.75, 1.0029)	(−0.0147, 0.75, 0.9969)
	*θ*	77.112	77.752	76.698	76.081	75.260	73.583	72.869

In addition, the orientation of the CH_3_NH_3_^+^ ion can be seen in the orthogonal CH_3_NH_3_PbI_3_. Table 1 shows the Cartesian coordinates of the C and N atoms are displaced by 0.01 Å along the [111] direction, which is similar to a past report that Pb and apical I atoms are displaced by 0.1 Å and 0.01 Å along the [001] direction in P4mm CH_3_NH_3_PbI_3_ (ref.^22^). The Pb-N-C angle decreases with increasing external electric field, which consistents with the increase in the lattice parameters and cell volume.

### Optical properties of CH_3_NH_3_PbI_3_ under an external electric field

**Figure 8:**
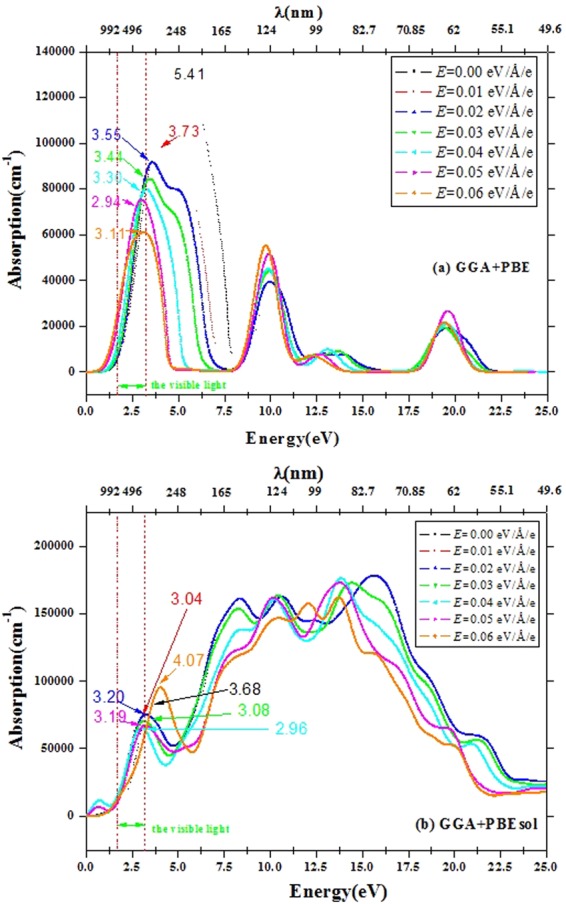
The optical absorption spectrum within the polarized with polarization (1, 0, 0) for CH_3_NH_3_PbI_3_ with the external electric field *E* = 0.00–0.06 eV/Å/e.

CH_3_NH_3_PbI_3_ may show different preferential growth directions with different substrates, so obtaining the optical performance in this growth direction is an important physical problem. Let us take the [100] direction as the preferred growth direction as an example to illustrate this problem. Figure 8 (a) calculated by GGA + PBE and (b) calculated by GGA + PBEsol show the optical absorption spectrum with polarized light, where the polarization is along the [100] direction of CH_3_NH_3_PbI_3_ in the presence of an external electric field. It is seen that the electric field significantly influences the optical absorption characteristics of CH_3_NH_3_PbI_3_. We assume the highest intensity absorption peak as the main absorption peak. The values 5.5 eV and 3.68 eV in the range of 0~5 eV for CH_3_NH_3_PbI_3_ calculated by the GGA + PBE and GGA + PBEsol methods are the positions of the main absorption peak without the external electric field, which corresponds to absorbed light with the highest frequency. As the external electric field increases, the peak positions shift to lower frequencies, approaching the visible light region, and full width at half maximum (FWHM) of the absorption peaks decreases, which makes light absorption more effective and thus improves the photoelectric conversion. In addition, there are some absorption peaks at higher energies beyond the visible range, which do not play a major role in photoelectric conversion, but can become a candidate as optical detection device such as ultraviolet band.

As the first-order approximation, the band gap *E*_g_ and wavelength should satisfy the following relation:

1 $${\lambda }({\rm{nm}})=1239.8/{E}_{{\rm{g}}}({\rm{eV}}).$$

We can obtain the related data based on eq. 1, as shown in Table 2. The following can be deduced:

(i) The band gap decreases non-linearly with increasing external electric field. When *E* ≤ 0.03 eV/Å/e, *ν and ν*_100_ decrease with an increase in the external electric field; when *E* ≥ 0.04 eV/Å/e, *ν and ν*_100_ increase with external electric field increasing, except *ν* with 0.04 eV/Å/e based on the GGA + PBE. Here, *ν* is the frequency calculated by the band gap, *ν*_100_ is the frequency along the [100] direction based on the Fig. 8.
(ii) The band gap (*E*g) based on the band structure is similar to *E*_g_’ based on the optical properties. Based on adiabatic approximation and single electron approximation, the relationship between absorption coefficient and photon energy can be expressed as,

2 $$\alpha =B(h\nu -E^{\prime} {}_{g}),$$

where *a* is the absorption coefficient, *B* is a fitting parameter, *h* is Planck’s constant, *v* is frequency, and *E*_g_′ is the band gap. According to the data in Fig. 8, *E*_g_′ values based on the optic properties are shown in Table 2.

**Table 2:** Related data obtained using eq (1). Here, *E* is the external electric field*, E*_g_ is the band gap based on the band structure, *λ* is the wavelength, *ν* is the frequency calculated by the band gap, *ν*_100_ is the frequency along the [100] direction based on the Fig. 8, and *E*_g_′ is the band gap based on the optical properties.

Functions	*E* (eV/Å/e)	*E*_g_ (eV)	*λ* (nm)	*ν* (10^8^MHz)	*ν*_100_ (eV)	*E*_g_′ (eV)
GGA + PBE	0.00	1.700	729.29412	4.11357	5.41	1.72
	0.01	1.658	747.7684	4.01194	3.73	1.63
	0.02	1.567	791.19336	3.79174	3.55	1.55
	0.03	1.587	781.22243	3.84014	3.44	1.57
	0.04	1.437	862.76966	3.47717	2.30	1.43
	0.05	1.213	1022.09398	2.93515	2.94	1.20
	0.06	1.073	1155.452	2.59639	3.11	1.06
GGA + PBEsol	0.00	1.675	740.1791	4.05307	3.68	1.68
	0.01	1.254	988.67624	3.03436	3.04	1.25
	0.02	1.266	979.3049	3.0634	3.20	1.24
	0.03	1.200	1033.16667	2.90369	3.08	1.19
	0.04	1.405	882.41993	3.39974	2.96	1.40
	0.05	1.330	932.18045	3.21826	3.19	1.34
	0.06	1.254	988.67624	3.03436	4.07	1.25

Moreover, although the preferred growth direction may be not [100] in practice, we provided a method to obtain the optical properties of CH_3_NH_3_PbI_3_ under an external electric field.

### Why does the band gap decrease non-linearly?

For CH_3_NH_3_PbI_3_ under an external electric field, the Hamiltonian can be written as,

3 $$\hat{H}={\hat{H}}_{0}+\hat{H}^{\prime} ,$$

Here, ${\hat{H}}_{0}=-\,\frac{{\hslash }^{2}}{2m}{\nabla }^{2}+V(r)$; $\hat{H}^{\prime} =e\overrightarrow{E}\cdot \overrightarrow{r}=eEr\,\cos \,\theta$; $e=1.6\times {10}^{-19}{\rm{C}}$; *E* is the external electric field; and *θ* is the angle between the direction of the electric field and the radius vector direction $\overrightarrow{r}$. $\hat{H}^{\prime}$ is the perturbation. Based on the perturbation theory, the energy levels (*E*_*i*_) will change to the initial values (*E*^(0)^_i_) by an amount *∆E*_*i*_.

4 $${E}_{i}={{E}^{(0)}}_{i}+{\rm{\Delta }}{E}_{i},$$

*∆E*_*i*_ can be expanded with the additional energy of the electric field, which are Stark effect orders denoted as first order *∆E*^(1)^_*i*_ or second order *∆E*^(2)^_*i*_.

5 $${\rm{\Delta }}E={\rm{\Delta }}{E}^{(1)}+{\rm{\Delta }}{E}^{(2)}+\cdots ,$$

The energy changes owing to the electric field, where <ψ_*i*_ | is the initial state of the system, then we have,

6 $${\rm{\Delta }}{E}^{(1)}=\langle {\psi }_{i}|-e\overrightarrow{E}\cdot \overrightarrow{r}|{\psi }_{i}\rangle =\langle {\psi }_{i}|-eEr\,\cos \,\theta |{\psi }_{i}\rangle ,$$

For second-order energy changes, the summation is over all possible states of the system, so,

7 $${\rm{\Delta }}{E}^{(2)}=\sum _{allstate{s}_{k}}\frac{\langle {\psi }_{i}|-e\overrightarrow{E}\cdot \overrightarrow{r}|{\psi }_{k}\rangle \langle {\psi }_{k}|-e\overrightarrow{E}\cdot \overrightarrow{r}|{\psi }_{i}\rangle }{{{E}^{(0)}}_{i}-{{E}^{(0)}}_{k}},$$

If *E* can be considered a constant over the perturbation volume,

8 $${\rm{\Delta }}{E}^{(1)}=-\,e\overrightarrow{E}\langle {\psi }_{i}|\overrightarrow{r}|{\psi }_{i}\rangle ,$$

9 $${\rm{\Delta }}{E}^{(2)}=\overrightarrow{E}\cdot \sum _{allstate{s}_{k}}\frac{\langle {\psi }_{i}|-e\overrightarrow{r}|{\psi }_{k}\rangle \langle {\psi }_{k}|-e\overrightarrow{r}|{\psi }_{i}\rangle }{{{E}^{(0)}}_{i}-{{E}^{(0)}}_{k}}\cdot \overrightarrow{E}.$$

**Figure 9:**
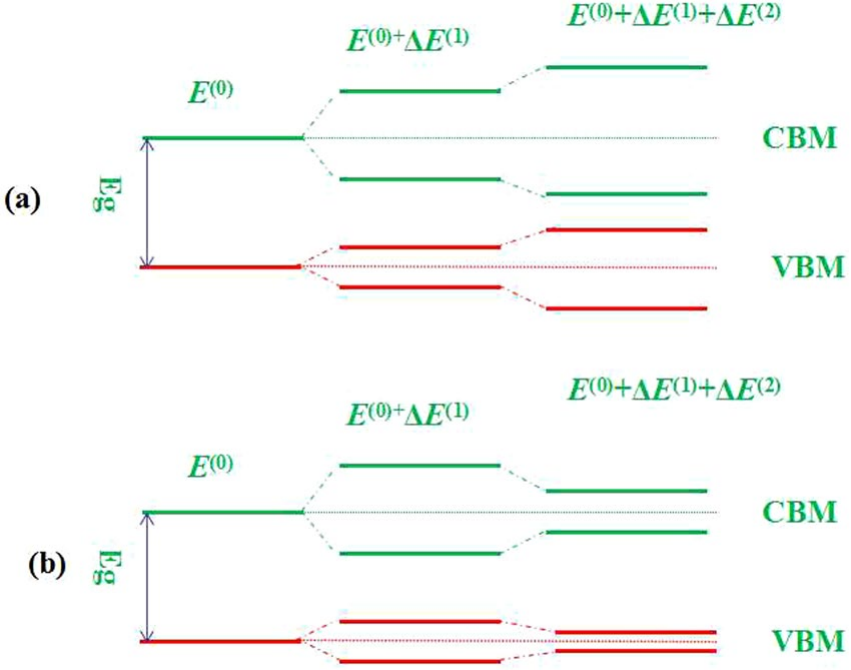
Schematic of the Stark effects on the band gap (*E*_g_) process, (**a**)*ΔE*^(2)^ > 0 and (**b**) *ΔE*^(2)^ < 0.

From Table 1, it can be seen that the movements of the I^−^ anions are coupled to the movements of the monovalent MA^+^ cations and the rotation of the MA dipoles. This change in polarizability in the domains can influence the second-order Stark effect through the change in the dielectric constant owing to the change in the optical absorption spectrum^28^. Then, *ΔE*^(2)^ may reflect in two possible first-order Stark effects, as shown in Fig. 9. If the value of *ΔE*^(2)^ is positive, the band gap will widen; if the value of *ΔE*^(2)^ is negative, the band gap will shrink. However, the second-order Stark effect cannot be larger than the first-order Stark effect, so CH_3_NH_3_PbI_3_ without an external electric field has the maximum band gap. The non-linear extent of the second-order Stark effect cause a non-linear change in the band gap.

**Figure 10:**
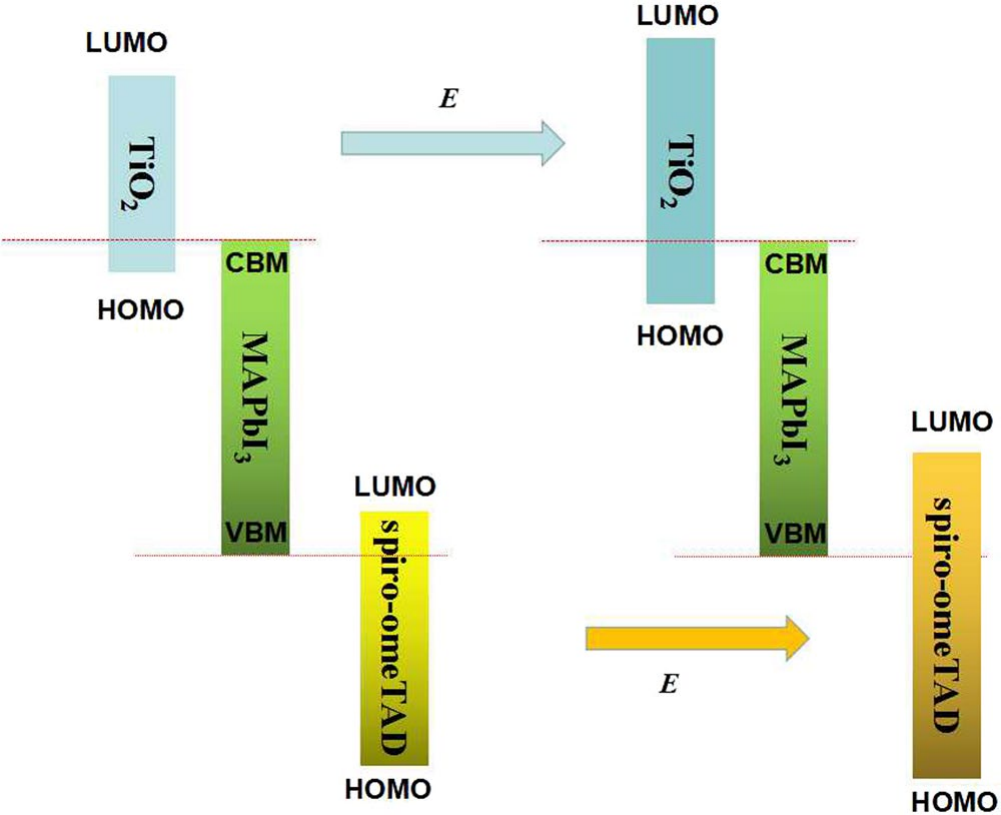
Schematic of external electric field on the LUMO and HOMO of TiO_2_ and spiro-omeTAD process.

The device required that: (i) The Highest Occupied Molecular Orbital (HOMO) of TiO_2_ as ETL layer must be lower than CBM of perovskite active layer. (ii) The Lowest Unoccupied Molecular Orbital (LUMO) of spiro-omeTAD as HTL layer must be higher than VBM of perovskite active layer. The external electric field induced the Stark effect, splitting energy levels for TiO_2_ and spiro-omeTAD (shown in Fig. 10), which decreases the HOMO of TiO_2_ and increases the LUMO of spiro-omeTAD.

### How to obtain the external electric field in practice?

As a preliminary exploration, we believe that this study is valuable. Indeed, the calculated results are aimed for the orthorhombic structure and not the tetragonal structure at room temperature, but the orthorhombic MAPbI_3_ structure can be applied to solar panels in space with lower temperature. Due to cosmic microwave background with 3 K^29^, the perovskite solar cells with orthorhombic phase can accomplish as Power generation device candidate in space such as International Space Station, satellite, space shuttle, spacecraft, lunar rover vehicle, etc. Moreover, this may provide a new idea to control the properties of the tetragonal structure at room temperature.

The main reason for the orthorhombic to tetragonal transition in CH_3_NH_3_PbI_3_ is temperature, and the external electric field causes the Stark effect splitting energy level of C, N, H, Pb and I, and the two structures with the same element and the similar chemical bond characters. Thus, we inferred that the Stark effect for CH_3_NH_3_PbI_3_ with a tetragonal structure may be observed at room temperature.

**Figure 11:**
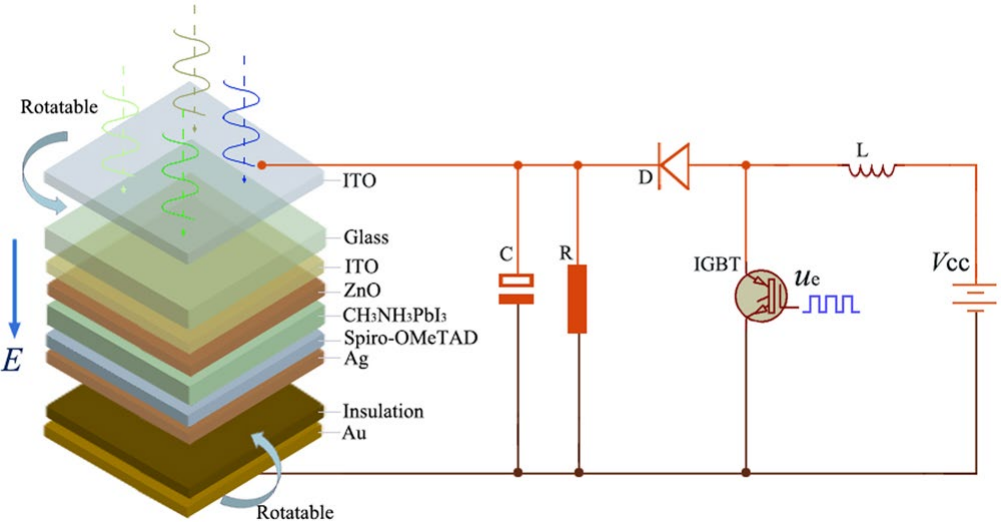
Device for realizing an external electric field. Here, *V*_CC_ is input voltage obtained by the storage battery charged by the solar battery, *L* is inductance, *u*_e_ is rectangular square wave pulse signal which control the duty cycle and regulate magnification, IGBT is crystal oscillator, *D* is diode, *R* is resistance, and *C* is capacitance.

The device for realizing the external electric field is shown in Fig. 11, which is similar to the ref. reported by Li *et al*.^30^. It noted that the device in this study only provide the external electric field, do not provide electrons. The large DC voltage near 100 V can be obtained by DC boost circuit, which can resolve the electric field strength. The positive electrode is connected with the transparent ITO for visible light, and the negative electrode is connected with Au; the directions of the two electrodes can control the direction of the electric field, including the [111] direction. Moreover, the distance between the positive and negative electrodes, as well as the insulation thickness and dielectric constant, influenced the electric field strength.

It noted that the external electric field of about 0.06 eV/Å/e is large, which is equal to 0.6 V/nm, but this external electric field can be carried out in practice. For example, Hsu *et al*. used an external magnetic field to adjust the relative energy levels between a skyrmion and a ferromagnet globally, and obtained an electric field of 1 V/nm, which corresponds to a magnetic field of about 40 mT for their system^31^. Qin *et al*. applied an external electric field to drive the ultra-low thermal conductivity of silicene. Using an electric field (*E*_z_ = 5 V/nm), the lattice thermal conductivity of silicene can be reduce a record low value of 0.091 Wm^−1^ K^−1^, which is comparable to that of the best thermal insulation materials^32^. The main problem caused by the huge electric field may be the device breakdown, which is attributed to the self-sustainable discharge for uniform electric field. The more uniform the electric field is, the higher the self-sustainable discharge voltage is. So the transparent ITO as the positive electrode and the Au film as the negative electrode should possess the rule shapes and flat surfaces, which would decrease the degree of dielectric polarization, and reduce the possibility of the tip discharge.

## Conclusion

In conclusion, we have used first-principles calculations to calculate the geometries, band structure, electronic properties, and optical absorption properties of perovskite CH_3_NH_3_PbI_3_ under an external electric field aligned along the [111] direction. The external electric field increases the lattice parameters and the cell volume, stretching the *c*-axis and influencing the degree of lattice distortion. The external electric field controls the band gap from 1.70 to 1.073 eV, corresponding to external electric field strengths of 0.00 to 0.06 eV/Å/e. The rotation of the MA dipoles induced a second-order Stark effect, and the non-linear change in the second-order Stark effect causes a non-linear change in the band gap. In addition, the absorption peaks and the peak locations are affected by the external electric field. Thus, this study provides a possible method to improve the photoelectric conversion of perovskite solar cells.

## Methods

**Figure 12:**
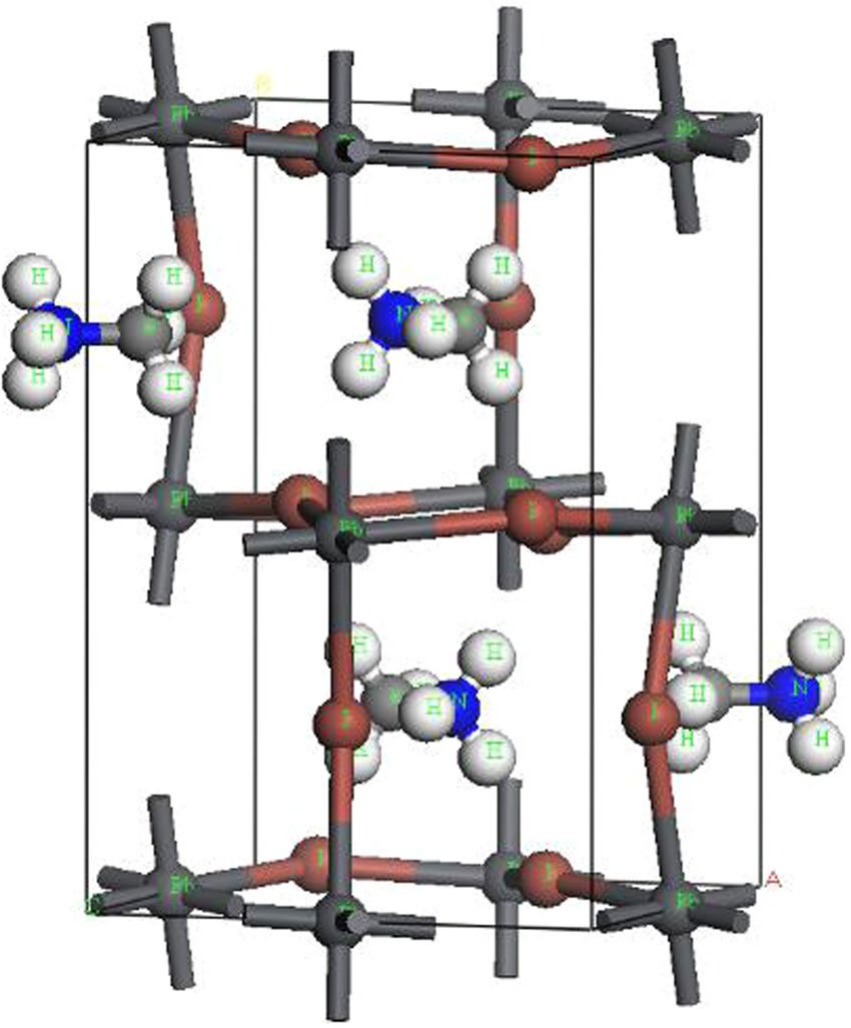
Unit cell of the orthorhombic CH_3_NH_3_PbI_3_ perovskite.Here, white, black, blue, gray, and brown balls are H, Pb, N, C and I atoms, respectively.

CH_3_NH_3_PbI_3_ perovskite undergoes two phase transitions, one at 160 K (orthorhombic to tetragonal) and the other at 330 K (tetragonal to cubic). The orthorhombic CH_3_NH_3_PbI_3_ structure could be closer to that at the 0 K, so we chose this as the research object given the DFT calculations are performed at 0 K. Based on the structure reported by Menéndez-Proupin *et al*.^25^, CH_3_NH_3_PbI_3_ perovskite has an orthorhombic crystal structure in the space group *Pnma* (no. 62), with lattice parameters of *a* = 8.8273 Å, *b = *12.6793 Å, and *c* = 8.5099 Å, as shown in Table 3 and Fig. 12. CH_3_NH_3_PbI_3_ has a typical AMX_3_ perovskite structure with the unit cell consisting of a central lead atom octahedrally coordinated to six iodide atoms. The PbI_6_ octahedron is located inside a cube with each iodide at the centre of a cubic face, and the CH_3_NH_3_ cations are positioned at the corners of the cube. To study the effect of the electric field on the physical properties of CH_3_NH_3_PbI_3_, the external electric field along different directions (*x*, *y*, and *z*) was investigated.

**Table 3:** The orthorhombic structure of CH_3_NH_3_PbI_3_ reported by Menéndez-Proupin *et al*.^25^.

Fractional coordinates			
Pb	0.00000	0.00000	0.50000
I	0.98041	0.25000	0.56319
I	0.18148	0.01821	0.17644
C	0.40839	0.25000	0.43168
N	0.55516	0.25000	0.51975
H	0.11881	0.31686	0.01029
H	0.15523	0.82116	0.96378
H	0.03804	0.25000	0.85812
H	0.93323	0.25000	0.19489

Space group 62 (Pnma).

Lattice parameters: *a* = 8.8273 Å, *b* = 12.6793 Å, *c* = 8.5099 Å.

We calculated the physical properties including the energy band, the density of states (DOS), and optical absorption in the orthorhombic perovskite CH_3_NH_3_PbI_3_ exposed to different external electric fields using the Cambridge Serial Total Energy Package (CASTEP)^33,34^ program. The generalized gradient approximation (GGA) functional developed by Perdew, Burke, and Ernzerhof (1996, 2008, PBE, and PBEsol)^23,24^ was used. These exchange-correlation functionals employed were same as those in the study of the self-regulation mechanism for charged point defects in hybrid halide perovskites reported by Walsh *et al*.^35^. The spin-orbit coupling (SOC) effect is reported to have little influence on the geometric structures^36^. Ultra-soft pseudopotentials with a cutoff energy of 310 eV (based on test results) were used to describe the interactions between the valence electrons and the ionic core, and including relativistic effects for Pb and I atoms. A 3 × 2 × 3 Monkhorst-Pack *k*-point scheme was used to calculate the absorption spectra. We performed the convergence test, and found the results with 500 eV cutoff energy and 5 × 5 × 5 Monkhorst-Pack *k*-point scheme to be similar to those with the above parameters. The convergence tolerances for geometry optimization calculations were set to a maximum displacement of 5.0 × 10^−4^Å, maximum force of 0.01 eV/Å, maximum energy change of 5.0 × 10^−6^ eV/atom, maximum stress of 0.02 GPa.

According to the refs^24,35^, PBEsol as exchange–correlation functional is a revision of the PBE functional, which improves equilibrium properties of densely-packed solids and their surfaces, specifically tailored for solids. It has been shown to yield structural data in accordance with experiment reported by F. Brivio *et al*.^37^. This functional predicts the structure of common London-dispersion corrected functions without the addition of an empirical potential.
